# Ovarian mitochondrial dynamics and cell fate regulation in an androgen-induced rat model of polycystic ovarian syndrome

**DOI:** 10.1038/s41598-020-57672-w

**Published:** 2020-01-23

**Authors:** Reza Salehi, Hannah L. Mazier, Anne-Laure Nivet, Arkadiy A. Reunov, Patricia Lima, Qi Wang, Arianna Fiocco, Ciro Isidoro, Benjamin K. Tsang

**Affiliations:** 1 Chronic Disease Program, Ottawa Hospital Research Institute, Ottawa, Canada; 2 Department of Cellular & Molecular Medicine and Obstetrics & Gynecology, University of Ottawa, Ottawa, Canada; 3 Electron Microscopy Laboratory, University of Ottawa Heart Institute, Ottawa, Canada; 4 Laboratory of Molecular Pathology, Department of Health Sciences, Università del Piemonte Orientale “A. Avogadro”, Via P. Solaroli 17, 28100 Novara, Italy

**Keywords:** Macroautophagy, Endocrine reproductive disorders, Endocrine reproductive disorders, Infertility, Endocrine reproductive disorders

## Abstract

In this study, we investigated in an androgenized rat model the involvement of autophagy and mitochondrial dynamics in granulosa cells in the pathogenesis of polycystic ovarian syndrome (PCOS) and its modulation by exogenous gonadotropin (eCG). We found 5α-dihydrotestosterone (DHT) treatment reduces ovarian length and weight with predominantly late antral and/or preovulatory stage follicles and no corpora lutea. DHT increased the population of large lysosomes (>50 micron) and macroautophagy, an event associated with granulosa cell apoptosis. Increased granulosa cell Dynamin Related Protein 1 (Drp1) content in the DHT group was accompanied by increased circular and constricted, but reduced rod-shaped, mitochondria. eCG eliminated all atypical follicles and increased the number of late antral and preovulatory follicles with less granulosa cell apoptosis. eCG-treated rats had a higher proportion of connected mitochondria, and in combination with DHT had a lower proportion of circular and constricted mitochondria than rats treated with DHT alone, suggesting that eCG induces mitochondrial fusion and attenuates fission in granulosa cells. In summary, we observed that DHT-induced up-regulation of Drp1 is associated with excessive mitochondrial fission, macroautophagy and apoptosis in granulosa cells at the antral stage of development in an androgenized rat model for PCOS, a response partially attenuated by exogenous gonadotropin.

## Introduction

Polycystic ovarian syndrome (PCOS) is a continuum of reproductive, endocrine and metabolic disorders and is characterized by suppressed granulosa cell proliferation, early antral follicle growth arrest, chronic anovulation, hyperandrogenemia, and insulin resistance^1,2^. These conditions affect up to 10% of women at reproductive age and accounts for 75% of anovulatory infertility^1,3^. Common symptoms that concern women with PCOS are hirsutism, acne and/or oily skin, irregular bleeding and infertility. Patients with PCOS have increased risk of long-term morbidities including type 2 diabetes mellitus, hypertension, cardiovascular diseases and various cancers^4^.

Dynamin related protein 1 (Drp1) is a cytoplasmic GTPase and is a key player in mitochondrial fission. During mitochondrial fission, Drp1 is recruited to the mitochondrial membrane and assembles an oligomer chain that coils around and constricts the mitochondrion to form two daughter mitochondria^5^. The activity of Drp1 is dependent on post-translational modifications, including site-specific phosphorylation^6–8^. In the human, when its Ser616 (Ser635 in rats) is phosphorylated by the cyclin-dependent kinase 1 (CDK1)/cyclin B complex, mitochondrial fission is initiated, whereas when Ser637 (Ser656 in rats) is phosphorylated by PKA, Drp1 function is inhibited and mitochondrial dynamics is shifted towards mitochondrial fusion^6,9^. Recent studies suggest that mitochondrial fission and fusion homeostasis is crucial for the maintenance of healthy follicular development. In *Drosophila* sp., Drp1-null follicle cells exhibited increased proliferation, resistance to apoptosis and developmental ovarian defects^10,11^. An oocyte-specific Drp1 knockout mouse resulted in multi-organelle aggregations and mitochondrial deformities in the oocyte as well as decreased granulosa cell proliferation, follicle growth arrest and anovulation^12^. However, to date, the examination of mitochondrial fission and fusion dynamics in granulosa cells in the context to PCOS has not been reported.

Autophagy, a cell survival mechanism involving the degradation of intracellular constituents, is mediated through three different pathways: macroautophagy, microautophagy and chaperone-mediated autophagy^13,14^. The main pathway is macroautophagy, which involves the assembly of a double membrane-bound vesicle (autophagosome) for the degradation of target proteins and membranes along with portion of cytoplasm. Macroautophagy (now on referred to as autophagy) can also perform selective autophagy of organelles, such as mitophagy, which involves the degradation of damaged mitochondria following mitochondrial fission^15^. Microautophagy and chaperone-mediated autophagy utilize the lysosome for the degradation of small portions of cytoplasm and of KFERQ-tagged cytosolic proteins via lysosomal membrane invagination and HSC70-mediated translocation, respectively^16,17^. Mitophagy and autophagic cell death have been reported to be associated with dysregulation in mitochondrial fission, granulosa cell death and follicular growth arrest in PCOS^18,19^. However, few studies using androgenized rodent models have determined the possible involvement of autophagy and its correlation with mitochondrial dynamics in the pathophysiology of PCOS.

Our recent studies have shown that 5α-dihydrotestosterone (DHT)-implanted rat PCOS model mimics many of the phenotypes of the human PCOS, including increased body weight gain and disrupted estrus cyclicity, thus allowing us to examine the molecular and cellular basis of PCOS^20–22^. Here, we employed this chronically androgenized rat model to determine if PCOS is associated with dysregulation in mitochondrial fission/fusion and autophagy, and to determine whether exogenous gonadotropin can modulate this dysregulation *in vivo*. We hypothesized that in PCOS a dysregulated increase in mitochondrial fission associated with mitophagy could lead to granulosa cell apoptosis followed by follicular growth arrest, responses that could be attenuated by gonadotropin. Indeed, here we show that up-regulation in Drp1 is associated with excessive mitochondrial fission and apoptosis in granulosa cells at the antral stage of follicular development and that these events were partially attenuated by exogenous gonadotropin.

## Results

We have previously shown that the ovary of a three-month DHT-induced PCOS rat model had a smaller size presented a higher number of structural abnormalities than that in control rats^20,23,24^. To examine the ovarian dysregulation and the possible regulatory mechanisms involved in PCOS with a shorter DHT treatment, an androgenized rat model (1-month DHT-implant) was investigated. Since controlled stimulation with gonadotropin is also known to ameliorate certain phenotypes of PCOS^23^, we also examined the possible influence of eCG (20 IU, i.p., 48 h) on a number of morphological and biochemical changes induced by DHT in the following *in vivo* experimental design: CTL, DHT, eCG and DHT + eCG.

### One-month DHT treatment reduces ovarian weight and disrupts estrus cyclity without systemic changes

**Figure 1:**
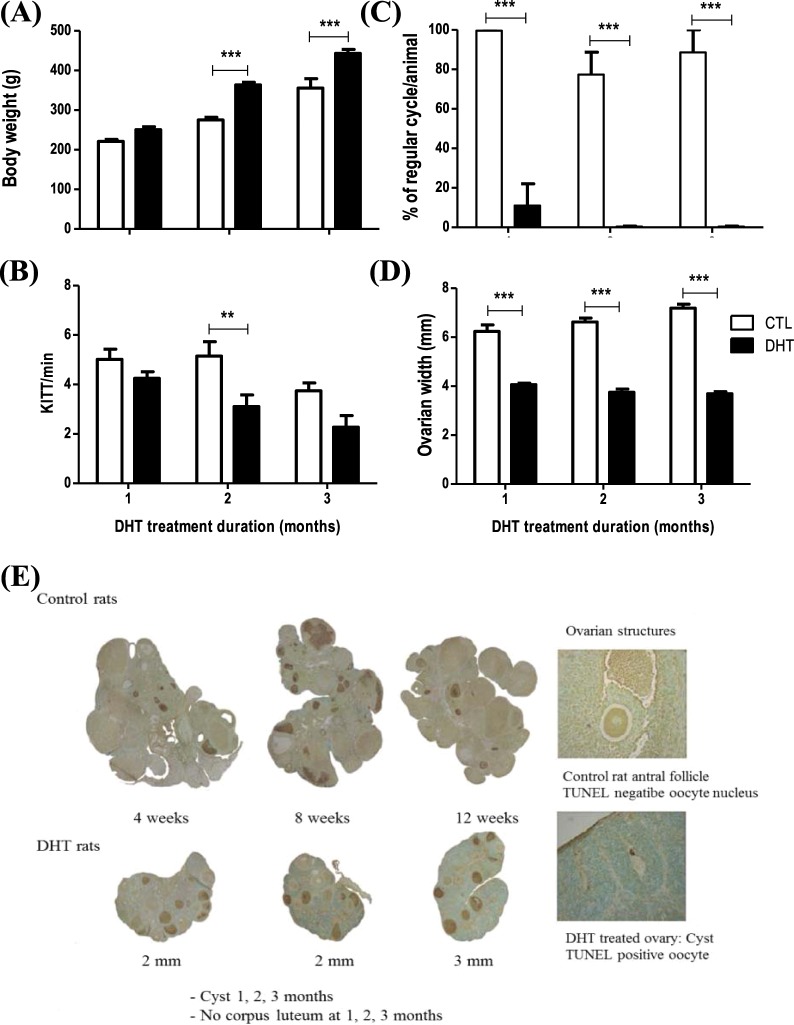
One-month DHT model demonstrates ovarian changes as in PCOS, but not the systemic changes. Rats were surgically implanted with sham control capsule or DHT (83 μg/d) time-release capsules for 1-, 2- or 3- month. (**A**) DHT treatment significantly increases body weight in 2 and 3 months, but not in 1 month (n = 8 rats per experimental group at each time point); (**B**) insulin sensitivity index significantly reduces in 2-month model, but not in 1- and 3-month models (n = 8 rats per experimental group at each time point); (**C**) DHT treatment dysregulates the estrus cycle in 1-, 2- and 3-month DHT treated rat model (n = 3 rats per experimental group at each time point); (**D**) Ovarian width significantly reduces in 1-, 2- and 3-month DHT treated rat model (n=8 rats per experimental group at each time point); (**E**) TUNEL (brown) and Methyl green coloration of ovarian paraffin sections from 1-, 2-, 3-month control and DHT rats. Data are presented as mean ± SEM, and analyzed by two-way ANOVA and Bonferroni *post hoc* test. **P < 0.01; ***P < 0.001 *vs*. CTL.

Two- and three-month DHT treatment significantly increased body weight (Fig. 1A; Two-way ANOVA and Bonferroni *post hoc* test, P < 0.001 *vs*. CTL), however there were no differences evident between CTL and DHT in one-month model. Insulin sensitivity index significantly reduced in two-months model, but not in one- and three-month(s) model. DHT treatment disrupted estrus cyclicity (Fig. 1C; Two-way ANOVA and Bonferroni *post hoc* test, P < 0.001 *vs*. CTL) and reduced ovarian width (Fig. 1D; Two-way ANOVA and Bonferroni *post hoc* test, P < 0.001 *vs*. CTL) in all three DHT treated models. These results suggest that one-month DHT model alters the ovarian characteristics without systemic changes.

### Ovary size and structural features are altered in a one-month DHT-induced rat model

**Figure 2:**
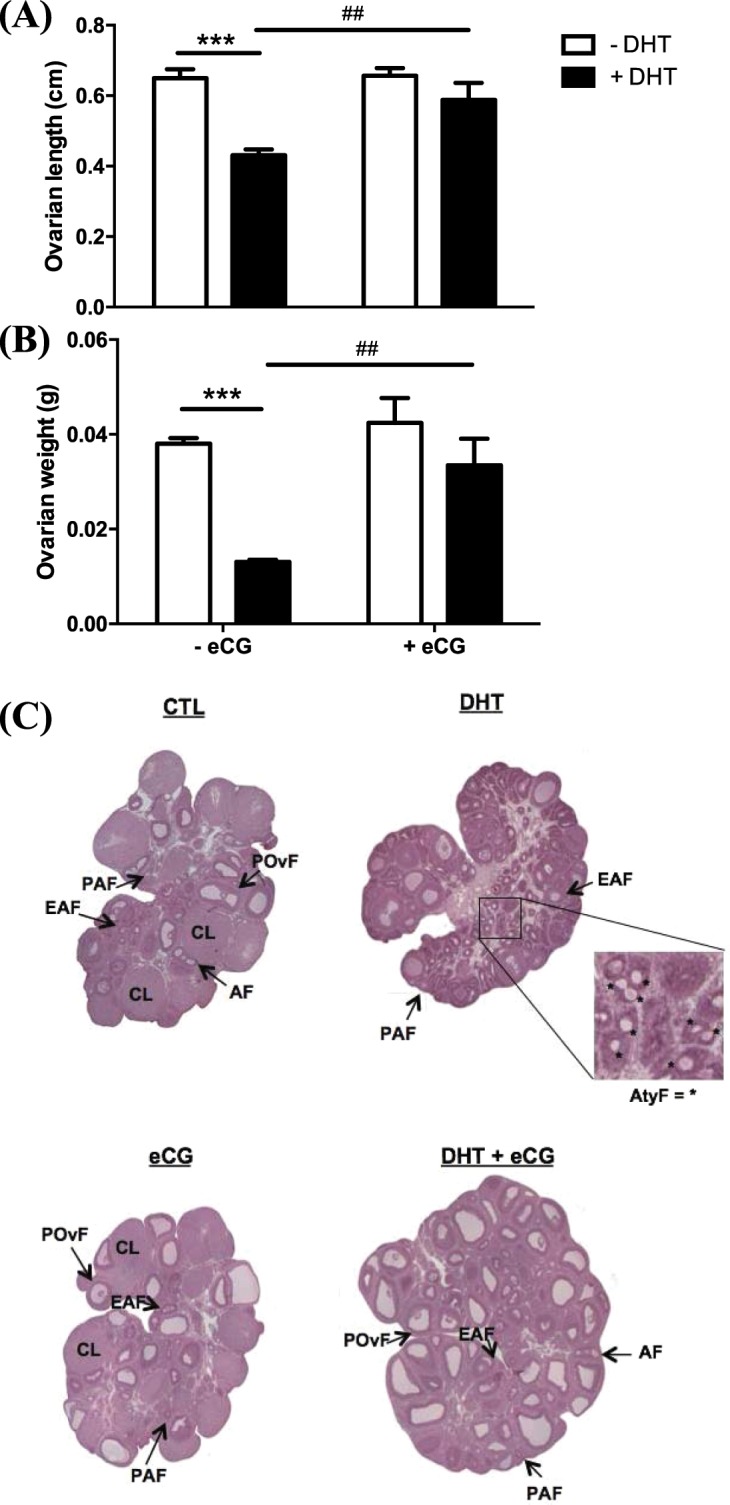
DHT reduced ovarian length and weight, responses reverted by gonadotropin injection. Rats were surgically implanted with sham control capsule or DHT (83 μg/d) time-release capsules for 1-month and injected with eCG (20 IU i.p./rat) 48 h prior to sacrifice. Fresh ovaries from each rat were measured for length (**A**) and weight (**B**). Data are presented as mean ± SEM of eight independent replicate experiments, and analyzed by two-way ANOVA and Bonferroni *post hoc* test. ***P < 0.001 *vs*. CTL. ^##^P < 0.01 *vs*. DHT alone.(**C**) representative sections of whole ovaries in each treatment group were visualized by H&E staining and variations in ovarian structural features were observed. PAF, preantral follicle; EAF, early antral follicle; POvF, preovulatory follicle; CL, corpus luteum; AtyF, atypical follicle.

DHT treatment resulted in a significant (Bonferroni *post hoc* test P < 0.001 *vs*. CTL) reduction of ovary length (Fig. 2A; DHT, P < 0.001) and weight (Fig. 2B; DHT, P < 0.001). H&E staining of ovary sections revealed important structural abnormalities in DHT-induced PCOS model (Fig. 2C). Compared to the wide range of follicular stages and corpora lutea observed in the ovaries of control rats, the ovaries of DHT-treated rats exhibited a high number of preantral, early antral and very few large follicles with no corpora lutea. The most distinctive feature in DHT ovary was the accumulation of many atypical follicles, which are described as small structures predominantly composed of theca cells with no discernible oocyte^20^.

### Exogenous gonadotropin attenuates apoptosis in granulosa cells in DHT-induced PCOS

**Figure 3:**
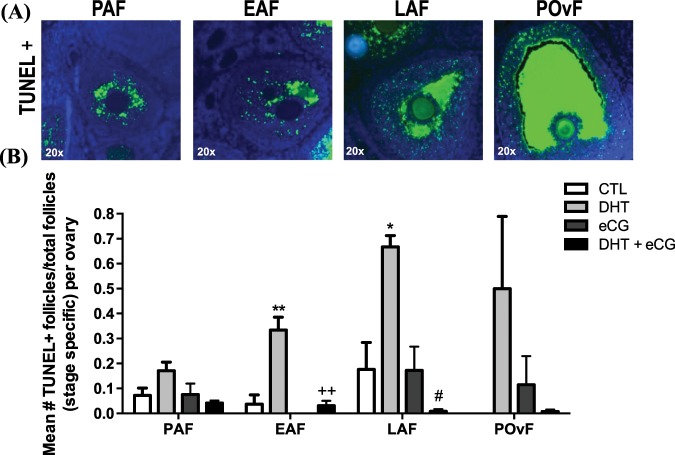
DHT induced granulosa cell apoptosis, an effect attenuated by gonadotropin. (**A**) Representative images of TUNEL positive (+) preantral (PAF), early antral (EAF), late antral (LAF) and preovulatory follicles (POvF) from a 1-month DHT-induced rat ovary. (**B**) After 1-month DHT implantation (83 μg/d) and 48 h after eCG injection (20 IU i.p./rat), granulosa cell apoptosis was determined in PAF, EAF, LAF and POvF of whole ovarian sections by fluorescent TUNEL assay, and was expressed as a ratio of mean number of TUNEL+ follicles over total ovarian follicles per rat* (*Rat = 1 ovary per rat/3 representative slides from whole ovary/3 sections per slide). Data are presented as mean ± SEM of three independent replicate experiments, and analyzed by two-way ANOVA and Bonferroni *post hoc* test. *P < 0.05; **P < 0.01 *vs.* CTL. ^#^P < 0.01 *vs.* DHT. ^++^P < 0.01 *vs.* DHT.

In search for the causes of follicular atrophy, we determined the occurrence of apoptosis in granulosa cells of the ovary at different stage of maturation (PAF, preantral follicle; EAF, early antral follicle; LAF, late antral follicle; POvF, preovulatory follicle) through a fluorescent TUNEL staining. As illustrated in Fig. 3A,B, DHT significantly increased granulosa cell apoptosis in early antral (Bonferroni: P < 0.01 *vs.* CTL) and late antral (Bonferroni: P < 0.05 *vs.* CTL) follicles. Interestingly, the administration of exogenous gonadotropin greatly prevented/inhibited apoptosis in DHT-treated ovaries (Fig. 3B).

### DHT increases morphological and molecular markers of autophagy

**Figure 4:**
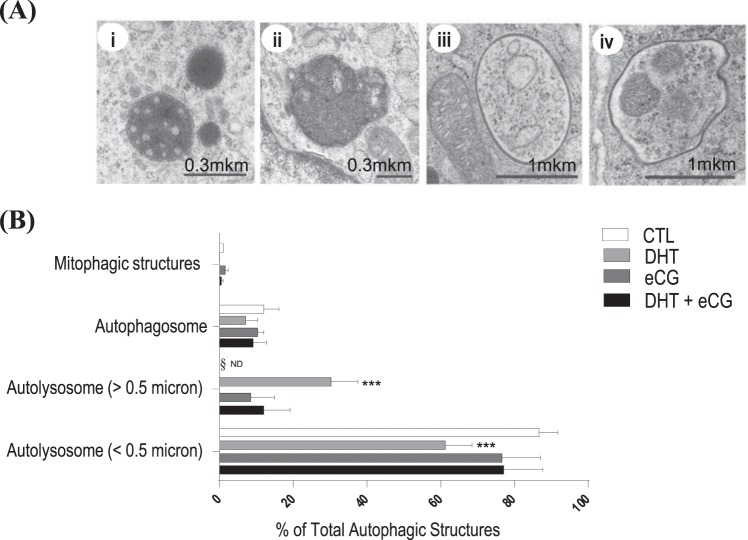
DHT treatment increased the population of large lysosomes (>50 micron). (**A**,i-iv) Representative examples of autophagic structures (autolysosome <0.5 micron, autolysosome >0.5 micron, autophagosome and mitophagic structures) using transmission electron microscopy (TEM) images. (**B**) After 1-month DHT implantation (83 μg/d) and 48 h following eCG (20 IU i.p./rat), TEM images of whole ovaries from each treatment group were captured and autophagic structures was assessed in granulosa cells. A minimum of 68 cells was assessed per treatment group. Data are presented as mean ± SEM of three independent replicate experiments, and analyzed by two-way ANOVA and Bonferroni *post hoc* test. ***P < 0.001 *vs*. CTL.

**Figure 5:**
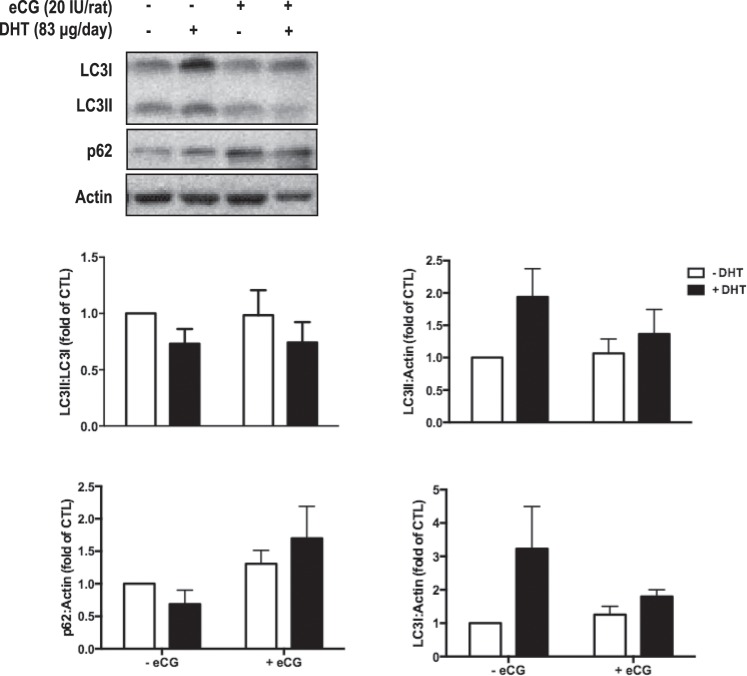
DHT treatment induces the accumulation of LC3 in Granulosa cells. After 1-month DHT implantation (83 μg/d) and eCG injection (20 IU/rat) 48 h prior to sacrifice, granulosa cells were isolated from rat ovaries and Western blots were performed to determine the protein content of LC3 (isoforms I and II) and of p62. Quantification of autophagy markers versus loading control as well as LC3II:LC3I ratio is represented in the histograms. Data are presented as mean ± SEM of six independent replicate experiments, and analyzed by two-way ANOVA and Bonferroni *post hoc* test.

Autophagic structures were examined by TEM in isolated granulosa cells from *in vivo* DHT-treated rats. TEM identified autolysosomes (Fig. 4Ai,ii), autophagosome with cytoplasmic content (Fig. 4Aiii) and with fragmented mitochondria (Fig. 4Aiv). The quantification indicated that DHT treatment increased the population of large autolysosomes (>50 micron, Two-way ANOVA and Bonferroni *post hoc* test, P < 0.001 *vs*. CTL, Fig. 4B) but not that of small autolysosomes (<50 micron). Noteworthy, eCG appeared to attenuate the increase in number of large-sized autolysosomes (Fig. 4B), although this was not statistically significant (P > 0.05). The presence of autophagosomes and mitophagic vesicles further supported the induction of autophagy in the PCOS granulosa cells (Fig. 4B). As a further confirmation, we assessed by Western blotting the conversion of LC3-I into LC3-II, indicative of autophagosome formation, and the expression of p62/SQSTM1, indicative of the autophagy degradation, in isolated granulosa cells. Data shown in Fig. 5 indicate that DHT increased the accumulation of LC3I (significantly, ANOVA P = 0.047) and trended towards to increase LC3II (P = 0.060) compared to CTL, along with a trended toward decreased accumulation of p62 (P > 0.05). Thus, though DHT had no significant effect on the absolute LC3II:LC3I ratio (two-way ANOVA: P > 0.05), these data consistently with TEM data suggest an induction of autophagy in DHT-treated samples.

**Figure 6:**
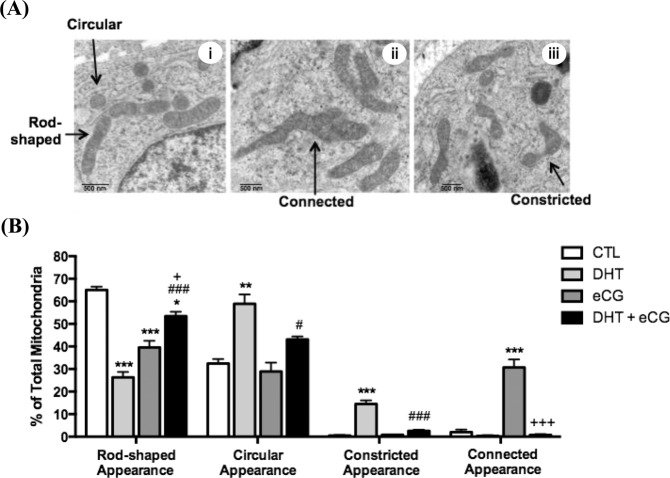
DHT-induced circular and constricted mitochondrial appearance were reduced by gonadotropin. (**A**, i–iii) Representative examples of mitochondrial phenotypes (rod-shaped, circular, constricted or connected appearance) in transmission electron microscopy (TEM) images. (**B**) After 1-month DHT implantation (83 μg/d) and 48 h following eCG (20 IU i.p./rat), TEM images of whole ovaries from each treatment group were captured and mitochondrial morphology was assessed. A minimum of 275 mitochondria was assessed per treatment group. Data are presented as mean ± SEM of three replicate experiments, and analyzed by two-way ANOVA and Bonferroni *post hoc* test. *P < 0.05, **P < 0.01, ***P < 0.001 *vs*. CTL; ^#^P < 0.05, ^###^P < 0.001 *vs*. DHT; ^+^P < 0.05, ^+++^P < 0.001 *vs*. eCG. Scale bar = 500 nm.

To examine the morphologic characteristics of mitochondria in the androgenized rat model, the ultrastructural morphology of granulosa cells in ovarian sections were examined by TEM (Fig. 6A). Quantification data in Fig. 6B indicates that DHT rats had fewer rod-shaped mitochondria (P < 0.001 *vs*. CTL) and more circular (P < 0.01 *vs*. CTL) and constricted (P < 0.001 *vs*. CTL) mitochondria compared to CTL rats, suggesting DHT treatment increased mitochondrial fission in granulosa cells.

**Figure 7:**
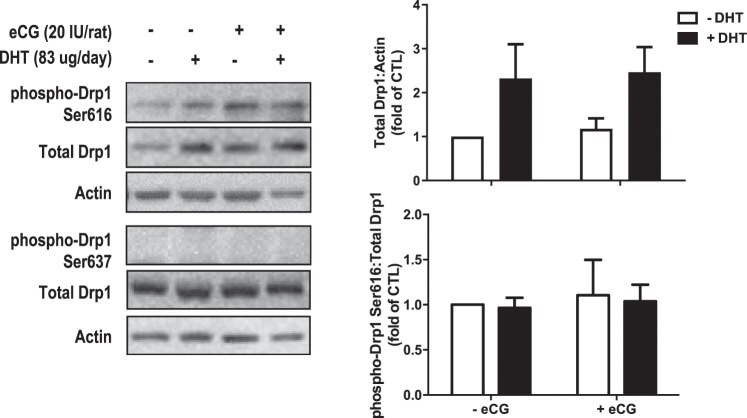
Granulosa cells in one-month DHT-treated rats exhibited increased total Drp1 content. After 1-month DHT implantation (83 μg/d) and 48 h after eCG (20 IU i.p./rat), granulosa cells were isolated and protein contents of phospho-Drp1 Ser616, phospho-Drp1 Ser637 and total Drp1 were assessed by Western blot. Data are presented as mean ± SEM of six independent replicate experiments, and analyzed by two-way ANOVA and Bonferroni *post hoc* test.

We also examined molecular indicators of mitochondrial fission in granulosa cells isolated from our *in vivo* treated rats. DHT significantly increased total Drp1 (Fig. 7, DHT, P = 0.016) while phospho-Ser616 content remained unaffected (DHT, P > 0.05) relative to total Drp1 (two-way ANOVA) and there were no observable bands for phospho-Drp1 Ser637. Bonferroni *post hoc* results were non-significant in phospho-Drp1 Ser616 and total Drp1.

### Gonadotropin attenuates the changes in ovarian morphology, granulosa cell apoptosis and mitochondrial morphology induced by DHT

In addition to studying the ovarian changes induced by DHT *in vivo*, we also investigated the possible modulatory role of gonadotropin on these ovarian responses. As shown in Fig. 2, two-way ANOVA indicate eCG treatment alone and in combination with DHT significantly increased ovarian length (Fig. 2A; eCG, P = 0.013; eCG × DHT, P = 0.021 *vs*. DHT) and weight (Fig. 2B; eCG, P = 0.003; eCG × DHT, P = 0.048 *vs*. DHT). Ovaries exposed to eCG alone had a higher number of corpora lutea and late antral/preovulatory follicles compared to both DHT alone or with eCG (Fig. 2C), suggesting that these animals have ovulated. eCG also appeared capable of eliminating all atypical follicles and reactivating folliculogenesis in DHT-treated animals, as evident by the high number of late antral and preovulatory follicles. DHT + eCG rats had less granulosa cell apoptosis compared to DHT rats in both early antral and late antral follicles (Bonferroni: P < 0.01 *vs*. DHT; Fig. 3B). However, no corpora lutea were present in the DHT-treated rats following eCG injection.

Autophagic structures and markers were also assessed in granulosa cells of *in vivo* samples, as shown in Figs. 4B and 5. eCG had no significant effects on autophagic structures (Two-way ANOVA, P > 0.05), LC3II:LC3I ratio (eCG, P > 0.05; eCG × DHT P > 0.05), LC3I content (eCG, P > 0.05; eCG × DHT P > 0.05), or LC3II content (eCG, P > 0.05; eCG × DHT P > 0.05). However, eCG significantly increased granulosa cell p62 content compared to DHT group (eCG, P = 0.033; eCG × DHT P > 0.05).

eCG-treated rats had a significantly higher proportion of connected mitochondria (P < 0.001 *vs*. CTL) compared to CTL rats, and a lower proportion of rod-shaped mitochondria (P < 0.001 *vs*. CTL). DHT + eCG rats had a significantly lower proportion of circular (P < 0.05 *vs*. DHT) and constricted (P < 0.001 *vs*. DHT) mitochondria compared to DHT rats alone (Fig. 6B), indicating eCG attenuates mitochondrial fission in granulosa cells. Despite these promising morphological results, Western blot analysis (Fig. 7) indicated that eCG had no significant effects on the contents of total Drp1 (eCG, P > 0.05; eCG × DHT P > 0.05) and phospho-Drp1 Ser616 (eCG, P > 0.05; eCG × DHT P > 0.05).

## Discussion

Although the etiology of PCOS remains elusive due to its complex and heterogeneous nature, granulosa cell apoptosis has been suggested to play an important role in follicular growth arrest associated with the pathogenesis of PCOS^20,25,26^. We demonstrated, for the first time, the dysregulation of mitochondrial fission and fusion dynamics, and autophagy in a DHT-induced rat model that recapitulates many phenotypes of human PCOS. We also showed the modulatory role of gonadotropin on granulosa cell mitochondrial dynamics in both physiological and pathophysiological conditions, as well as the mechanisms involved. These findings highlight the importance of mitochondrial dynamics in the granulosa cell, and how dysregulation in the regulatory processes of cell death may be involved in the pathogenesis of PCOS. The present studies improve our current understanding of the regulatory roles of gonadotropins in granulosa cell fate decision and the potential cellular mechanism of therapeutic action for women with PCOS.

A common approach in studying the cellular and molecular basis of PCOS is the utilization of animal models^27,28^. Although no “perfect” animal model currently exists for human PCOS due to its complex nature, the androgenized rat model has proven to be useful as it mimics many phenotypes of the human condition^20,22–24^. In the current studies, we used a DHT-induced rat model with a shorter (one month) duration of treatment and assessed its efficacy by examining ovary size, ovarian structural features, and granulosa cell apoptosis. Similar to the three-month DHT-induced rat model and human PCOS^20,25,26,29^, in one-month DHT-induced rat, we found an induction of early antral granulosa cell apoptosis, follicular growth arrest, disrupted folliculogenesis and anovulation, but without systemic changes (e.g. body weight changes and insulin sensitivity index). We also observed the accumulation of small atypical follicles and a reduction in ovarian length and weight, features that are characteristic of the fifteen days^21^ and three-month DHT-induced model^20,23^. In a previous study^21^ where fifteen days DHT-induced model was used, fifteen days of DHT treatment reduces the ovarian weight with a tendency to decrease the number of pre-ovulatory follicles, and increase in early antral and unhealthy follicles. Reduced ovarian length and weight were consistent with previous results from 15d, 2 and 3 months of DHT treatment. Together, these results suggest that reduced the ovarian size results from decreased the number of large antral follicles, a phenotype which were reversed by eCG injection (DHT *vs* DHT + eCG groups), as indicated in Fig. 2C. One-month DHT treatment provides a more convenient and less expensive model for studying ovarian responses independent of systemic influences in PCOS, and that we can reliably use this model to perform novel studies pertaining to the pathogenesis anovulatory infertility in of PCOS^21^.

Autophagy acts first and foremost as a cell survival mechanism by recycling damaged intracellular contents and preventing cellular damage and death^13,14,30^. Its dysregulation, which is often associated with the induction of apoptosis and excessive mitochondrial fission, can lead to the pathogenesis of disease^15,31,32^. As mitochondrial fission and apoptosis are implicated in our DHT-induced model, it is possible that autophagy in granulosa cells plays a role in the pathogenesis of PCOS. In the present study, we assessed autophagic structures by TEM and Western blot analysis of the autophagy markers LC3 and p62 in the ovarian granulosa cells of rat PCOS model. We used LC3 and p62 western blotting along with TEM to monitor autophagy since when assessed together these techniques are considered the most reliable ones to assess macroautophagy, whatever the pathway in place for autophagosome formation^33^. In fact, non-canonical autophagy pathways in which autophagosome formation may initiate even in the absence of autophagy proteins (e.g., ATG5 or ATG7) have been reported^34,35^.

We found that DHT treatment increased the population of large lysosomes (>50 micron) and the cellular content of LC3 along with trended reduction of p62, indicating increased autophagy. A major challenge in assessing autophagy by TEM and molecular methods is its rapid and highly dynamic nature. It may be difficult to catch the appropriate time-of-action to see an effect when investigating a quick or dynamic process, and this is especially true during *in vivo* studies with a chronic DHT-induced model^36,37^. Taking together, TEM and molecular data can be interpreted as DHT mainly activates macroautophagy at the time of granulosa cell collection, with up-regulation of LC3 protein for building a higher number of autophagic vesicles, a picture reported in PCOS^38^.

Deregulated mitochondrial dynamics can lead to excessive mitochondrial fission and cell death in the pathogenesis of certain diseases^38–40^. To assess the potential role of mitochondrial dynamics in PCOS, we evaluated morphologic and molecular indicators of mitochondrial fission in our one-month DHT-induced rat model. The reduction in rod-shaped mitochondria implied a disruption of the normal, baseline state of mitochondrial dynamics in our DHT-induced model. The suppression of connected mitochondria by DHT alluded to a reduction in mitochondrial fusion events^41,42^. The higher proportion of circular and constricted morphologies suggested that excessive mitochondrial fission occurred in our DHT-induced model^41–43^. Overall, these morphologic and ultrastructural findings suggest that a substantially number of mitochondrial fission events occur in our DHT-induced rat model. The morphologic changes were associated with up-regulation of total Drp1 which likely reflect a compensatory demand for increased mitochondrial fission in our chronic DHT-treated model. Since the phosphorylated contents of Drp1 remained unaffected by DHT treatment, it is possible that chronic androgenization regulates Drp1 exclusively through modulation of total content and not on its phosphorylation. However, phosphorylation is a rapid process and it may not be possible to find an optimal time for these assessments in our chronic animal model. Our studies indicate that this excessive mitochondrial fission is associated with granulosa cell apoptosis and follicular growth arrest, which is consistent with its possible role in the pathogenesis of PCOS. These results are compatible with an earlier study demonstrating the synthetic androgen methyltrienolone increases total Drp1 contents and enhances mitochondrial fission and apoptosis in prostate cells^44^.

Gonadotropin is a key promoter of ovarian folliculogenesis^45–47^. A dysregulated increase in LH:FSH ratio, as evident in PCOS, could impair folliculogenesis and lead to antral follicle growth arrest and oligo-/anovulation^29,48^. Controlled gonadotropin therapy is a treatment strategy to reactivate folliculogenesis and ovulation in women with PCOS^49,50^. However, the modulatory role of gonadotropin in our DHT-induced rat model is not completely understood. In our current studies using one month androgenized rat model, we observed a restoration of ovarian length and weight, as well as morphological changes in the whole ovary but not some of the biochemical parameters following eCG injection in DHT-treated rats. However, it is possible that 1-month of DHT treatment induces a weaker PCOS phenotypes compared to 2 and 3 months models. Therefore, it might be difficult to detect the rescue effects of exogenous eCG in 1 month DHT treated rats compared to those seen in 2- and 3- month DHT treated rats.

The morphological changes induced by eCG in DHT-treated animals, including the elimination of atypical follicles and increase number of late antral and preovulatory follicles, implies the reactivation of folliculogenesis and follicular maturation. Although corpora lutea were not seen in our model to suggest the occurrence of ovulation, it is likely that the follicles were unresponsive to the LH-like capabilities of eCG and that the endogenous LH surge had not yet occurred^51^. Nonetheless, our results appear to correspond with the findings seen in the three-month DHT-induced model, indicating that eCG can effectively modulate DHT-induced changes after only one month of implantation. Furthermore, we found that eCG attenuated DHT-induced granulosa cell apoptosis at the early antral and late antral follicular stages, suggesting that the gonadotropin is anti-apoptotic in granulosa cells of PCOS, leading to the reactivation of folliculogenesis and the restoration of ovary size. There are limitations to our assessment of eCG modulation on autophagic structures and markers in our *in vivo* chronic DHT-rat model. We could not detect a significant effect of DHT and eCG in the conversion of LC3I to LC3II, probably because the time-of-action for which might have been missed during our assessment in the chronically androgenized modal *in vivo*. However, we could document that eCG attenuated the accumulation of large autolysosomes while increasing the accumulation of p62.

Our findings that eCG up-regulates p62 content are consistent with the notion that the gonadotropin inhibits autophagic flux, leading to suppression of autophagy. However, the assessment of autophagy by other experimental approaches is required to determine the effective role of autophagy in the etiology of PCOS^52^.

Our studies on mitochondrial morphology demonstrate that eCG reduced the proportion of circular and constricted mitochondria in DHT-induced rats, and increased the proportion of rod-shaped (normal) mitochondria. The restoration of DHT-induced changes in mitochondrial dynamics suggests a modulatory role of FSH at the mitochondrial level. The inability of eCG to alter the phosphorylated Drp1 contents *in vivo* was unanticipated. A possible reason for this could be due to its very rapid response and escape detection in a chronic model system. These findings do not exclude the possible involvement of other mitochondrial dynamics regulators (e.g. Fis1, Mfn1/2, Opa1), which will be the subject of future investigations.

**Figure 8:**
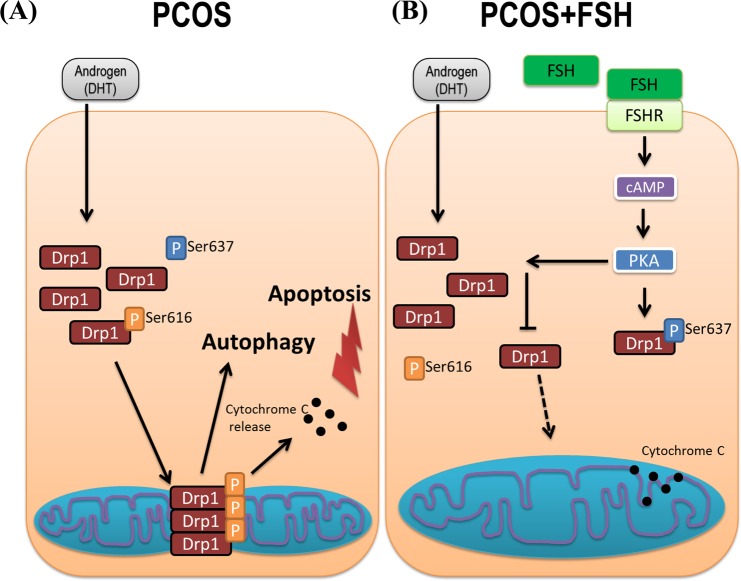
Hypothetical model of showing androgen excess-induced changes in granulosa cell mitochondrial dynamics in PCOS and the influence of gonadotropic stimulation. (**A**) In PCOS, androgen excess results in the up-regulation of Drp1, leading to increased mitochondrial fission. This dysregulation in mitochondrial fission results in increased autophagy and apoptosis, leading to early antral follicular growth arrest; (**B**) Gonadotropin stimulation attenuates mitochondrial fission, apoptosis and follicular growth arrest in PCOS.

Using a rat PCOS model, we have demonstrated that the involvement of mitochondrial dynamics in the pathogenesis of PCOS as well as its regulation by gonadotropin (e.g. eCG). To facilitate future investigation on mitochondrial dynamics in folliculogenesis and its deregulation in PCOS we hereby propose the following hypothetical model (Fig. 8). In PCOS (Fig. 8A), androgen excess results in the up-regulation of Drp1 leading to overabundant increased mitochondrial fission. This dysregulation in mitochondrial fission results in an increased in apoptosis and possibly autophagy, leading to early antral follicular growth arrest. Gonadotropin stimulation (Fig. 8B) suppressed mitochondrial fission, apoptosis and follicular growth arrest in PCOS. We observed, for the first time, that up-regulation in Drp1 was associated with excessive mitochondrial fission and apoptosis in granulosa cells at the antral stage of development in an androgenized rat model for PCOS, a response partially attenuated by exogenous gonadotropin. Our findings improve the current understanding of the pathobiology of PCOS.

## Material and Methods

### Reagents and antibodies

Cell culture media (M199), fetal bovine serum (FBS), penicillin and streptomycin, fungizone, trypsin, Trypan blue and ProLong® Gold Antifade Mountant with DAPI were purchased from Life Technologies (Burlington, ON, Canada). Equine chorionic gonadotropin (eCG), dimethyl sulfoxide (DMSO), anti-LC3 antibody and bovine serum albumin (BSA) were obtained from Sigma-Aldrich (Oakville, ON, Canada). Dihydrotestosterone (DHT) was provided from Steraloids Inc. (Newport, RI, USA), with prior approval from Health Canada, the Government of Canada. RBC lysis buffer was from eBiosciences (San Diego, CA, USA). Mouse monoclonal anti-rat Drp1 antibodies were purchased from Santa Cruz (Dallas, TX, USA). Mouse monoclonal anti-rat β-actin antibody was purchased from Abcam (Cambridge, MA, USA). Rabbit polyclonal anti-rat p62, phospho-Drp1 Ser637 and phospho-Drp1 Ser616 antibodies were obtained from Cell Signaling (Danvers, MA, USA). Horseradish peroxidase-conjugated secondary antibodies, reagents for DC™ Protein Assay and reagents for SDS-PAGE were purchased from Bio-Rad (Mississauga, ON, Canada). Enhanced chemiluminescence (ECL™) reagent, ECL Prime reagent and PageRuler™ Prestained Protein Ladder were purchased from Thermo Fisher Scientific (Burlington, ON, Canada). All other chemicals were of the highest analytical grade and were from Sigma-Aldrich.

### Animal preparation, estrus cycle and insulin sensitivity assessment

Female Sprague-Dawley rats (Charles River, Montréal, Canada) were maintained on 12-h light, 12-h dark cycles and given food and water *ad libitum*. All procedures were carried out in accordance with the Guidelines for the Care and Use of Laboratory Animals, Canadian Council on Animal Care, and were approved by the University of Ottawa Animal Care Committee.

The DHT-filled silicone capsules were prepared as previously described^53^. Briefly, rats at 21 d of age were implanted subcutaneously with either empty SILASTIC® capsules as sham control (CTL) or the capsule filled with DHT for continuous-release (83 µg/d) during one-month to mimic the hyperandrogenic state in women with PCOS^54,55^. At 26 d post-implantation, half the rats with CTL implants and half the rats with DHT implants were injected with eCG (20 IU, i.p.) or saline 48 h prior to sacrifice, resulting in a total of four treatment groups: CTL, DHT, eCG, and DHT + eCG.

Rat ovaries were collected after euthanization via CO_2_ and ovarian weight/length were measured. One ovary in each rat was fixed in 4% paraformaldehyde, paraffin embedded and serial sectioned (5 µm) and mounted onto positively charged slides for evaluation of the ovarian structure (hematoxylin and eosin staining) and granulosa cell apoptosis (TUNEL assay). The second ovary was fixed in 2.5% glutaraldehyde in 0.1 M cacodilate buffer for conventional transmission electron microscopy (TEM)^56^. Standard procedures for dehydration and Spurr’s Low Viscosity embedding mixture (Electron Microscopy Sciences, Hatfield, PA, USA) were performed as per manufacturer’s instructions. Sections were contrasted with toluidine blue (Electron Microscopy Sciences) for follicle-stage selection, and micrographs were taken on a JEOL 1230 transmission electron microscope. Estrous cycle and insulin sensitivity of rats were assessed as described previously^23^.

### Isolation of granulosa cells

Granulosa cells from CTL, DHT, eCG or DHT + eCG rats were collected as previously described^53^, carefully avoiding puncture of preovulatory follicles and corpora lutea. Granulosa cells were then washed and centrifuged in PBS and followed by treatment with red blood cell lysis buffer (Cell Signaling^®^, USA) for 1 min. After washing by centrifugation (800 × g, 5 min) in PBS again, cell pellets were immediately stored at −80 °C for protein extraction.

### Assessment of ovarian structures and categorization of follicle stages

To categorize follicles at different stages of development, we assessed the presence of the antrum, the number of granulosa and theca cell layers, the presence of mural and cumulus granulosa cells, and relative follicle size^57^. The presence of corpora lutea was also noted. Only follicles that had visible oocytes were categorized. Primary and secondary follicles had a single- and double-layer of granulosa cells, respectively. Preantral follicles had a minimum of 3 layers of granulosa cells with no antrum. Early antral follicles had a small antrum, taking up less than half of the entire follicle size. Late antral follicles had a substantial antrum that took up at least half of the follicle. Preovulatory follicles had the largest antrum taking up most of the follicle, and had even layers of mural granulosa cells as well as cumulus granulosa cells surrounding the oocyte. Atypical follicles were small collapsed structures in which an oocyte was not detectable, and had predominantly theca cells.

### Apoptosis assessment

*In situ* TUNEL assay was carried out in ovarian sections using TUNEL enzyme (#11 767 305 001, Roche, Mississauga, ON, Canada) and fluorescent TUNEL label (#11 767 291 910, Roche, Mississauga, ON, Canada) as per the manufacturer’s instructions. Images were obtained (×20 objective) on a Zeiss® Axioplan 2 Imaging microscope, using Axiovision® Release 4.8.2 imaging software. Follicles with visible oocytes were categorized into preantral, early antral, late antral and preovulatory stages, and TUNEL positivity was determined if ≥50% of the granulosa cells had positive staining. TUNEL positivity was expressed per follicle stage as the mean number of TUNEL positive follicles over total follicles, with the mean of three representative slides per rat.

### Autophagy assessment

TEM images of whole ovaries from each treatment group (CTL, eCG, DHT and DHT + eCG) were captured and autophagic structures were identified in granulosa cells. Autolysosomes, an indicator of macroautophagy, were dark vesicles surrounded by one membrane. Autophagosomes were intracellular double-membrane vesicles containing cytoplasmic material or mitochondria (indicative of mitophagy). Each category of autophagic structures was expressed as a percentage of total autophagic structures assessed in each treatment group, and a minimum of 68 cells was assessed in each. Autophagy was further assessed by western blotting of the autophagy markers LC3 and p62 (see below), according to the published guidelines^33^.

### Assessment of mitochondrial morphology

After whole ovaries were imaged by TEM, mitochondrial morphology in granulosa cells from preantral/antral follicles was categorized as showing: (a) rod-shaped appearance; (b) circular appearance; (c) connected appearance or (d) constricted appearance. Mitochondria with rod-shaped appearance were thin and elongated not exceeding 1500 nm in length. The membranes and inner cristae structures of these mitochondria were clear and well-defined. Mitochondria with circular appearance had clearly outlined mitochondrial membranes, though the interior could have bright (unhealthy) areas and poorly defined inner cristae structures. Mitochondria with constricted appearance were “dumb-bell shaped”, with an area of reduced width that appears constricted or “pinched in” (non-uniform width). The membrane was still intact and clear across this region of constriction, but interior structures including cristae structures were not well defined and bright areas were common. Mitochondria with connected appearance had at least one of the following characteristics: (a) highly elongated with the length exceeding 1500 nm; (b) mitochondria that appeared “forked” or misshapen (non-rod); or (c) mitochondria that interacted either distally or laterally. The clarity of the membrane and interior structures of connected mitochondria was variable. Each category of mitochondria was expressed as a percentage of total mitochondria assessed per treatment group, and a minimum of 275 mitochondria was assessed per treatment group.

### Protein extraction and western blot

Total proteins were extracted by directly adding 100 µl lysis buffer [10 mM Tris, pH 7.4; 1% sodium dodecyl sulfate (SDS), 1 mM sodium orthovanadate] to granulosa cells of CTL, DHT, eCG and DHT + eCG groups. The extracts were then sonicated and centrifuged (12,000 × g, 5 min). Protein concentration was determined by BioRad DC® protein assay kit. Whole cell lysates (20 µg protein) were subjected to SDS-PAGE and proteins were electrophoretically transferred to a nitrocellulose membrane (GE Healthcare Lifesciences, Mississauga, ON, Canada).

After blocking with 5% skim milk in TBST (0.05% Tween-20 in 10 mM Tris; 0.15 M NaCl, pH 7.4; room temperature, 1 h), membranes were incubated overnight at 4 °C with antibodies against Drp1 (1:1000), phospho-Drp1 Ser616 (1: 1000), phospho-Drp1 Ser637 (1:500), LC3 (1:1000), p62 (1:500) in TBST with constant agitation. Membranes were then treated with 1:5000 horseradish peroxidase-conjugated rabbit or mouse secondary antibodies (room temperature, 1 h), washed three times with TBST, then visualized with enhanced chemiluminescence, according to the manufacturer’s instructions. The intensity of the immunoreactive bands, obtained by both CL-Xposure film (Thermo Fisher Scientific) and VersaDoc®, were determined by densitometric quantification by AlphaEaseFC (Alpha Innotech, San Leandro, CA, USA) and normalized to β-actin (1:10, 000) as loading controls (Supplementary information).

### Statistical analysis

As indicated in the figure legends, data are presented as mean ± SEM of a minimum of three independent experiments. Two-way ANOVA was performed using GraphPad Prism^®^ 6.0 statistical software (San Diego, CA, USA) and multiple comparisons were achieved by Bonferroni *post hoc* test. Significant difference was defined at P < 0.05, as indicated by “*”, “^+^” or “^#^”.

## Supplementary information

Supplementary figures.
