# Supplementary material for: Ovarian mitochondrial dynamics and cell fate regulation in an androgen-induced rat model of polycystic ovarian syndrome
Source: Sci Rep. 2020 Jan 23;10:1021. doi: 10.1038/s41598-020-57672-w (PMC6978404; doi:10.1038/s41598-020-57672-w)

Ovarian mitochondrial dynamics and cell fate regulation in an androgen-induced rat model of polycystic ovarian syndrome

Reza Salehi[1], Hannah L. Mazier[1, 2], Anne-Laure Nivet[1,2], Arkadiy A. Reunov[3], Patricia Lima[1], Qi Wang[1], Arianna Fiocco[2,4], Ciro Isidoro[4] and Benjamin K. Tsang[1, 2*]*

[1]Chronic Disease Program, Ottawa Hospital Research Institute, Ottawa, Canada
[2]Department of Cellular & Molecular Medicine and Obstetrics & Gynecology, University of Ottawa, Ottawa, Canada
[3] Electron Microscopy Laboratory, University of Ottawa Heart Institute, Ottawa, Canada
[4] Laboratory of Molecular Pathology, Department of Health Sciences, Università del Piemonte Orientale "A. Avogadro", Via P. Solaroli 17, 28100, Novara, Italy

Abbreviated title: Mitochondrial dynamics and cell death in PCOS

Keywords: Drp1, LC3, Polycystic ovarian syndrome, Androgen, Autophagy, Gonadotropin

*Co-corresponding authors:
Professor Benjamin K. Tsang
The Ottawa Hospital – General Campus; Critical Care Wing - 3 rd Floor, Room W3107
501 Smyth Road, Mail Box #511, Ottawa, Ontario K1H 8L6
Tel: 1-613-798-5555, Ext. 72926 Email: btsang@ohri.ca

Professor Ciro Isidoro
Laboratory of Molecular Pathology and Nanobioimaging, Department of Health Sciences, Università del Piemonte Orientale "A. Avogadro", Via P. Solaroli 17, 28100, Novara, Italy.
Tel: +39 0321 660507 Email: ciro.isidoro@med.uniupo.it

Fig. 5

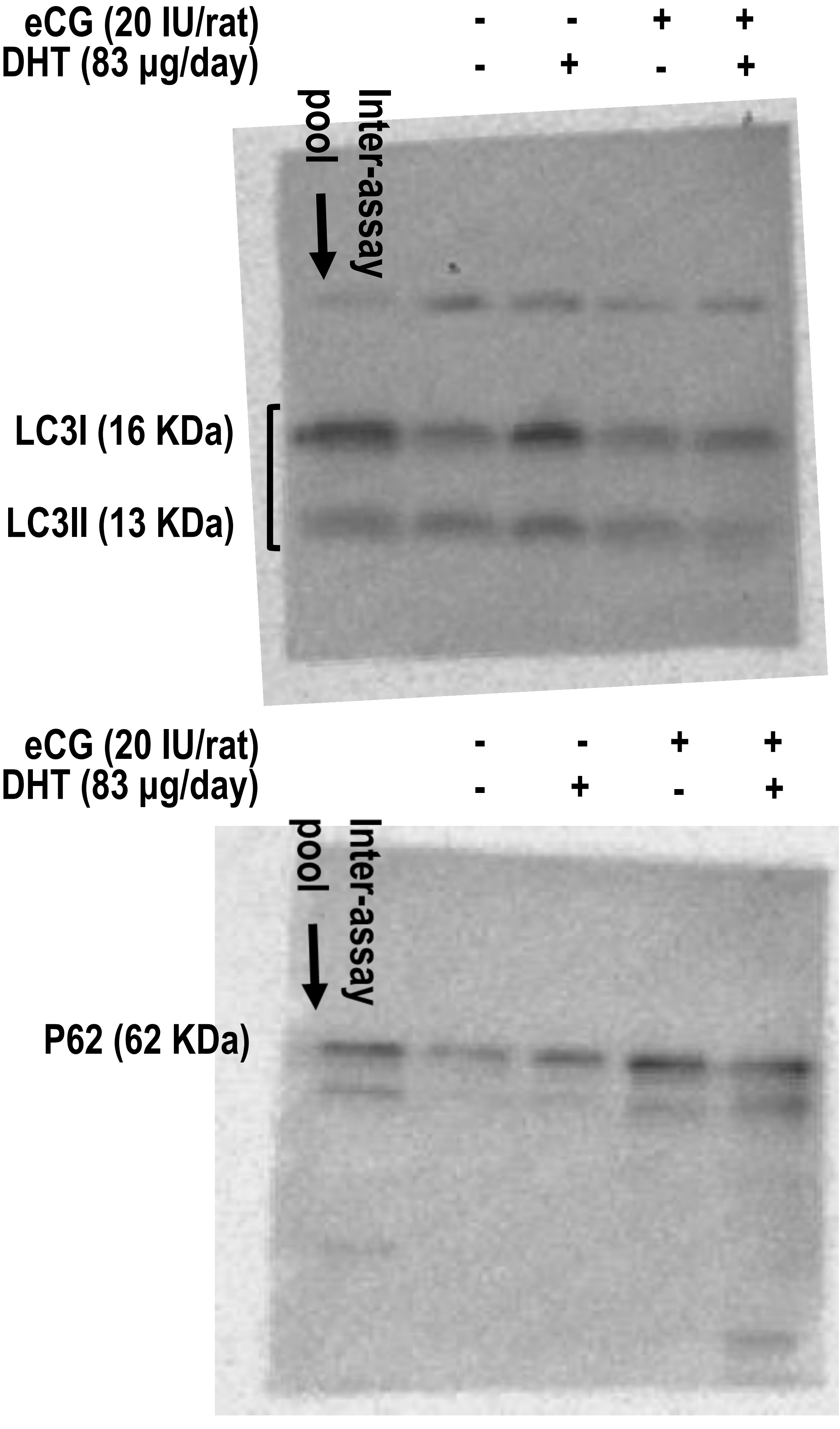
eCG (20 IU/rat) - - + +
DHT (83 µg/day) - + - +
Inter-assay pool
LC3I (16 KDa)
LC3II (13 KDa)
eCG (20 IU/rat) - - + +
DHT (83 µg/day) - + - +
Inter-assay pool
P62 (62 KDa)

Fig. 5

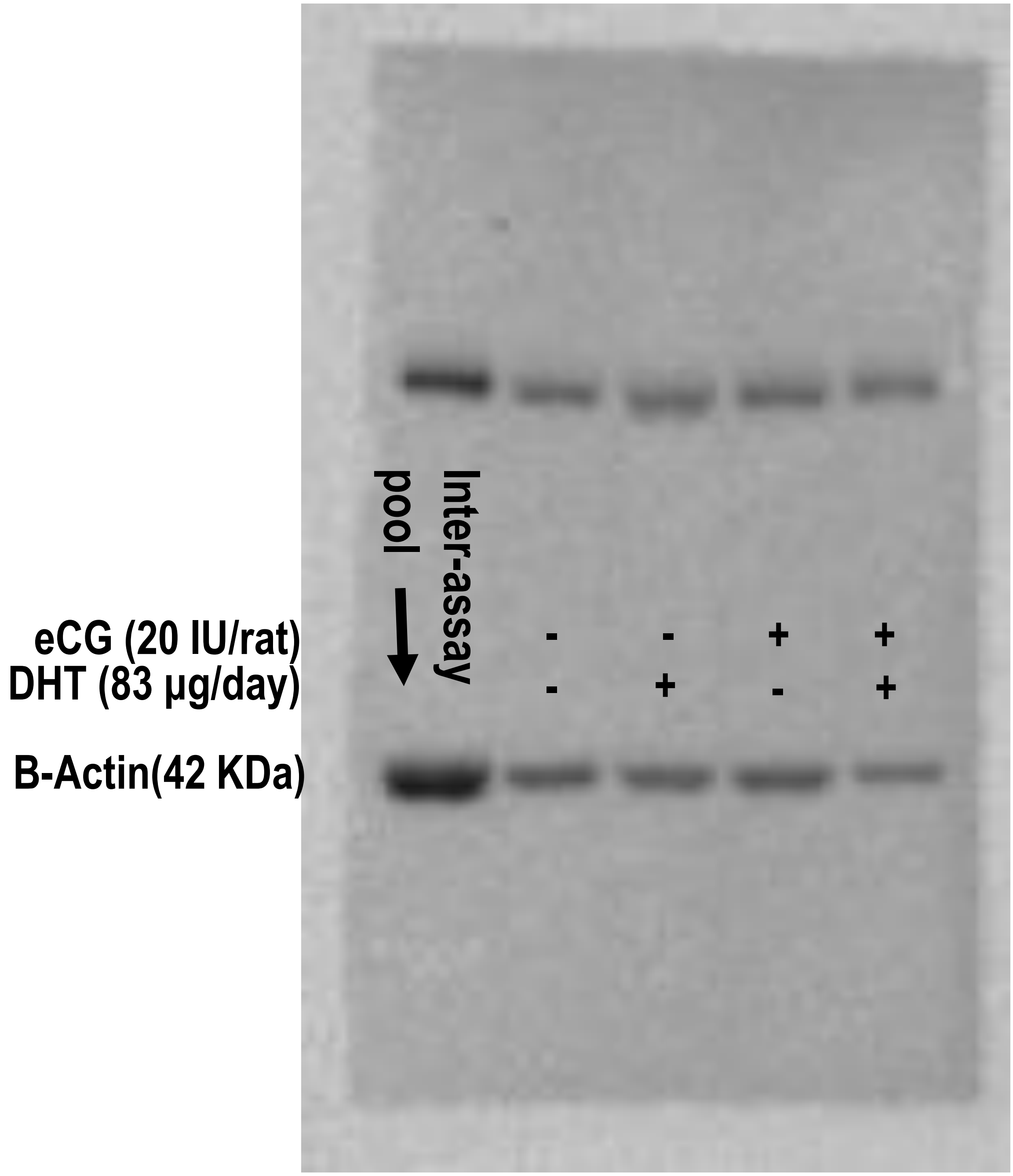

Fig. 7

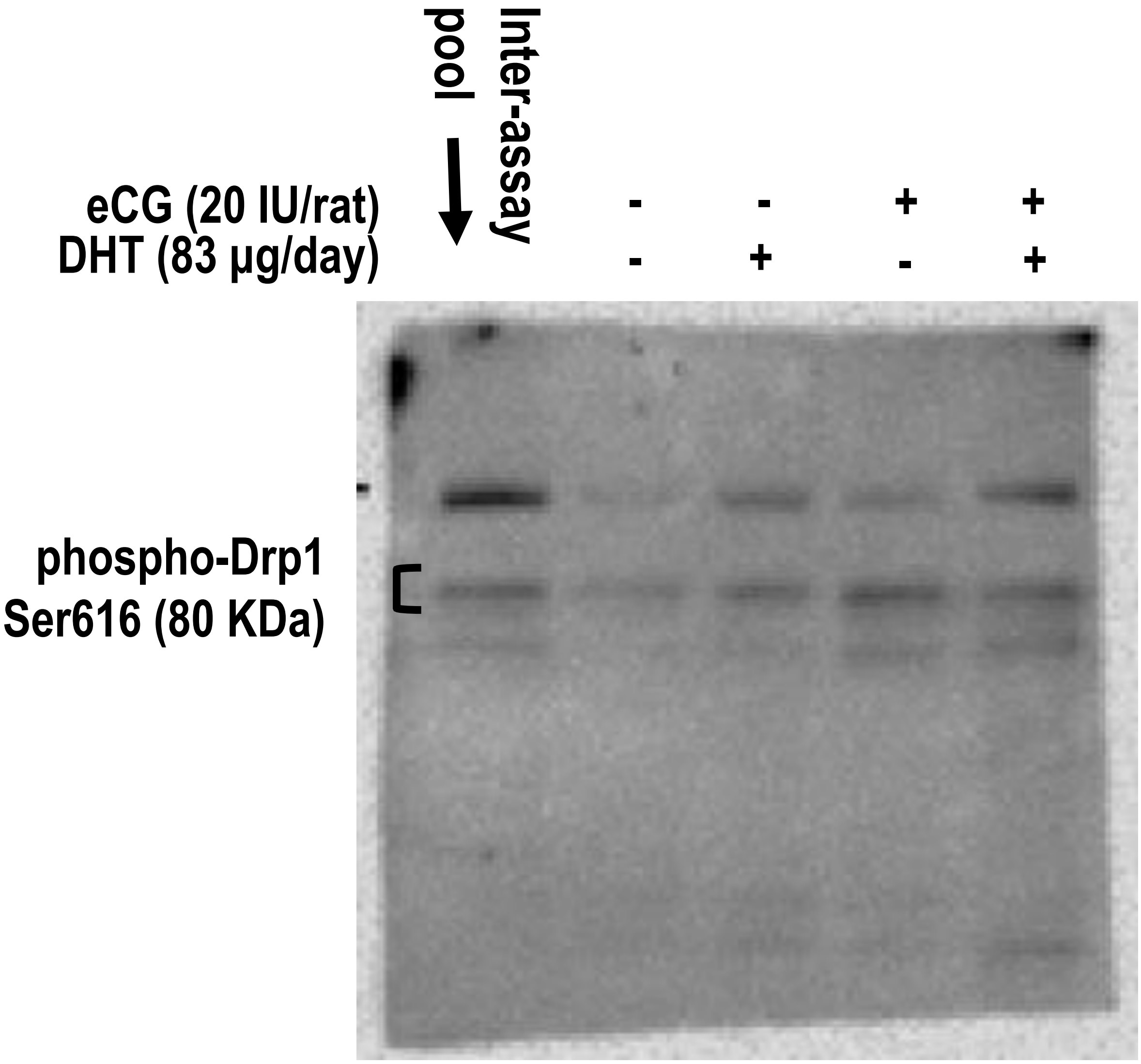
Inter-assay pool
eCG (20 IU/rat) - - + +
DHT (83 µg/day) - + - +
phospho-Drp1
Ser616 (80 KDa)

Fig. 7

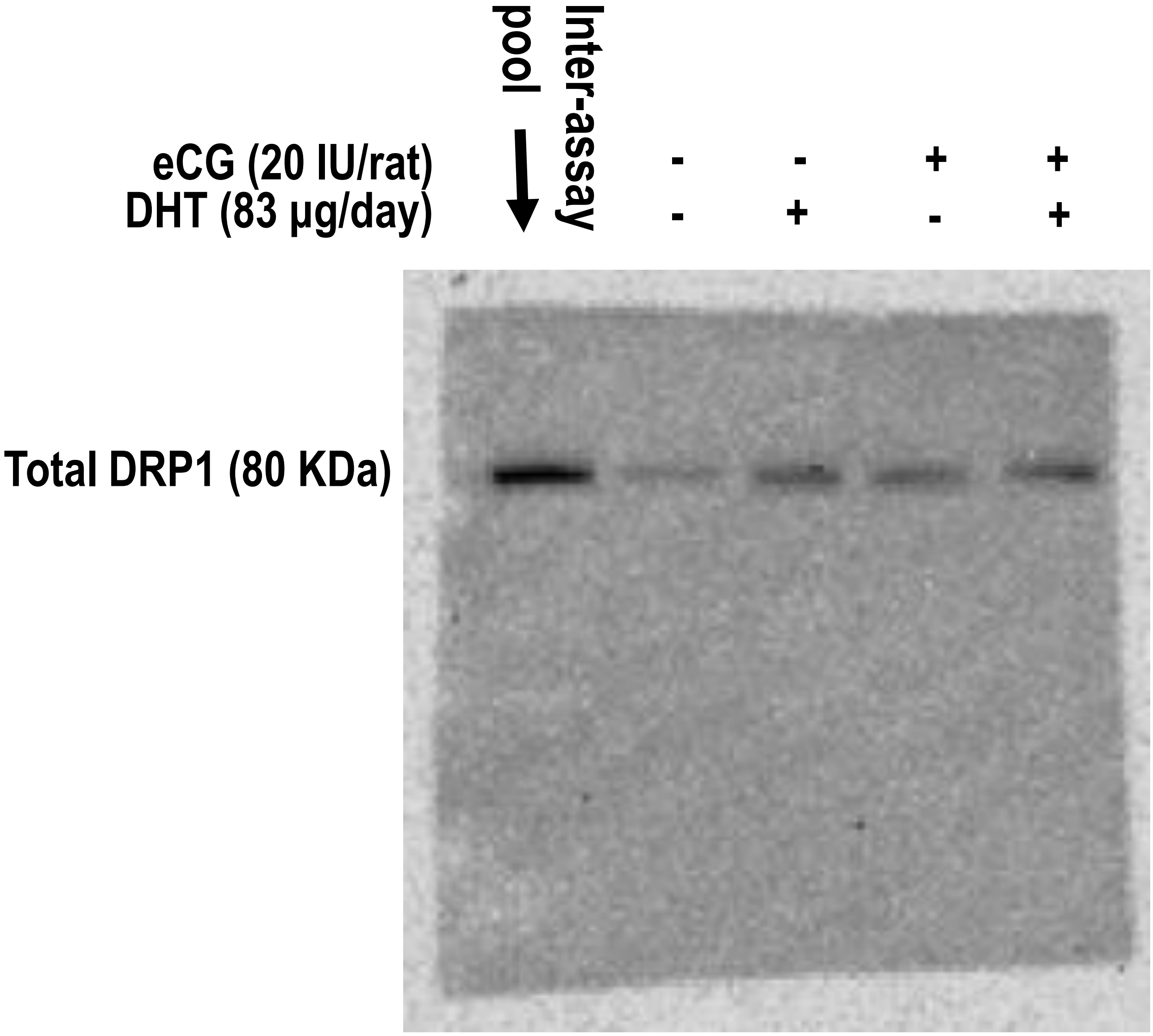

Fig. 7

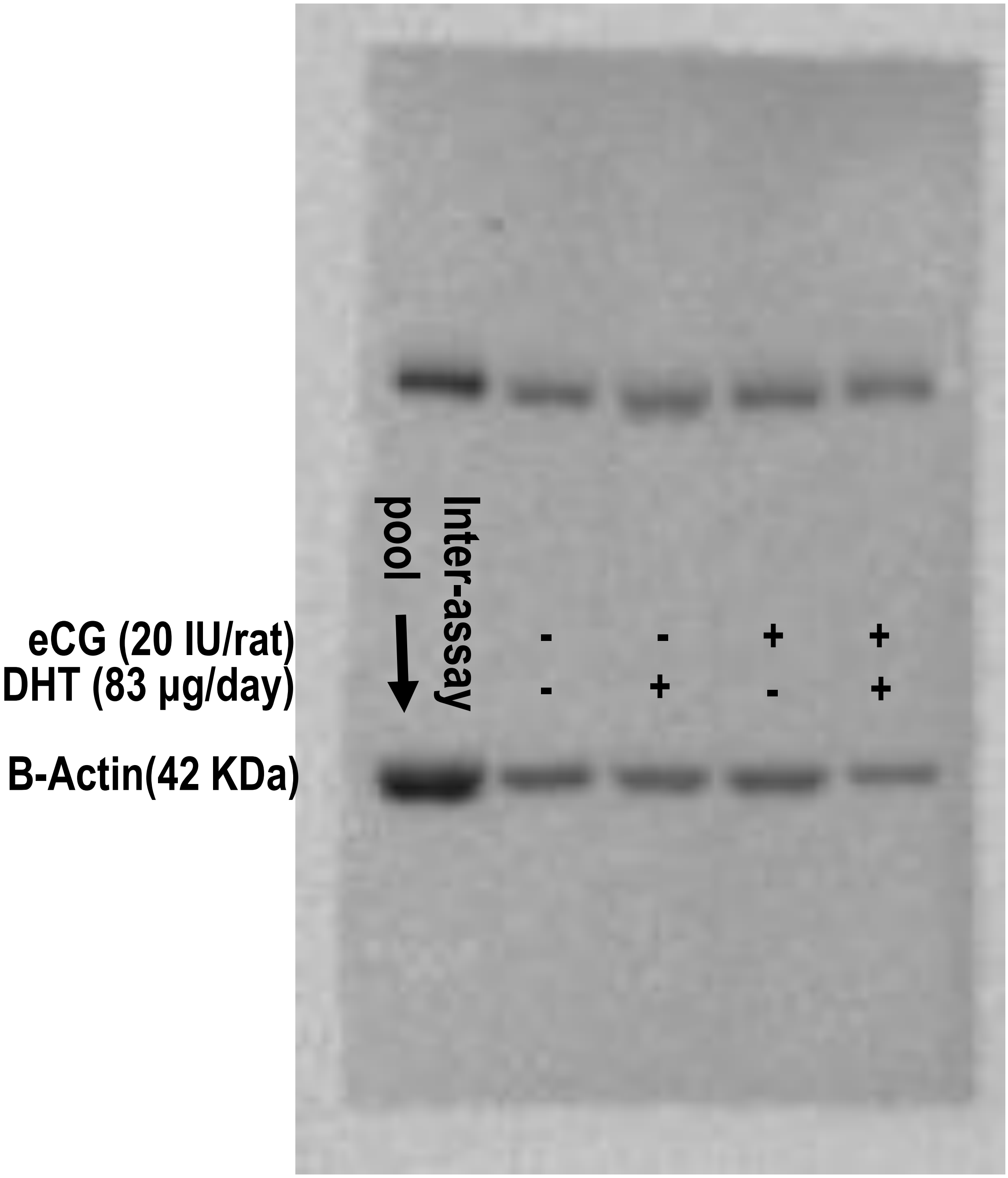

Fig. 7

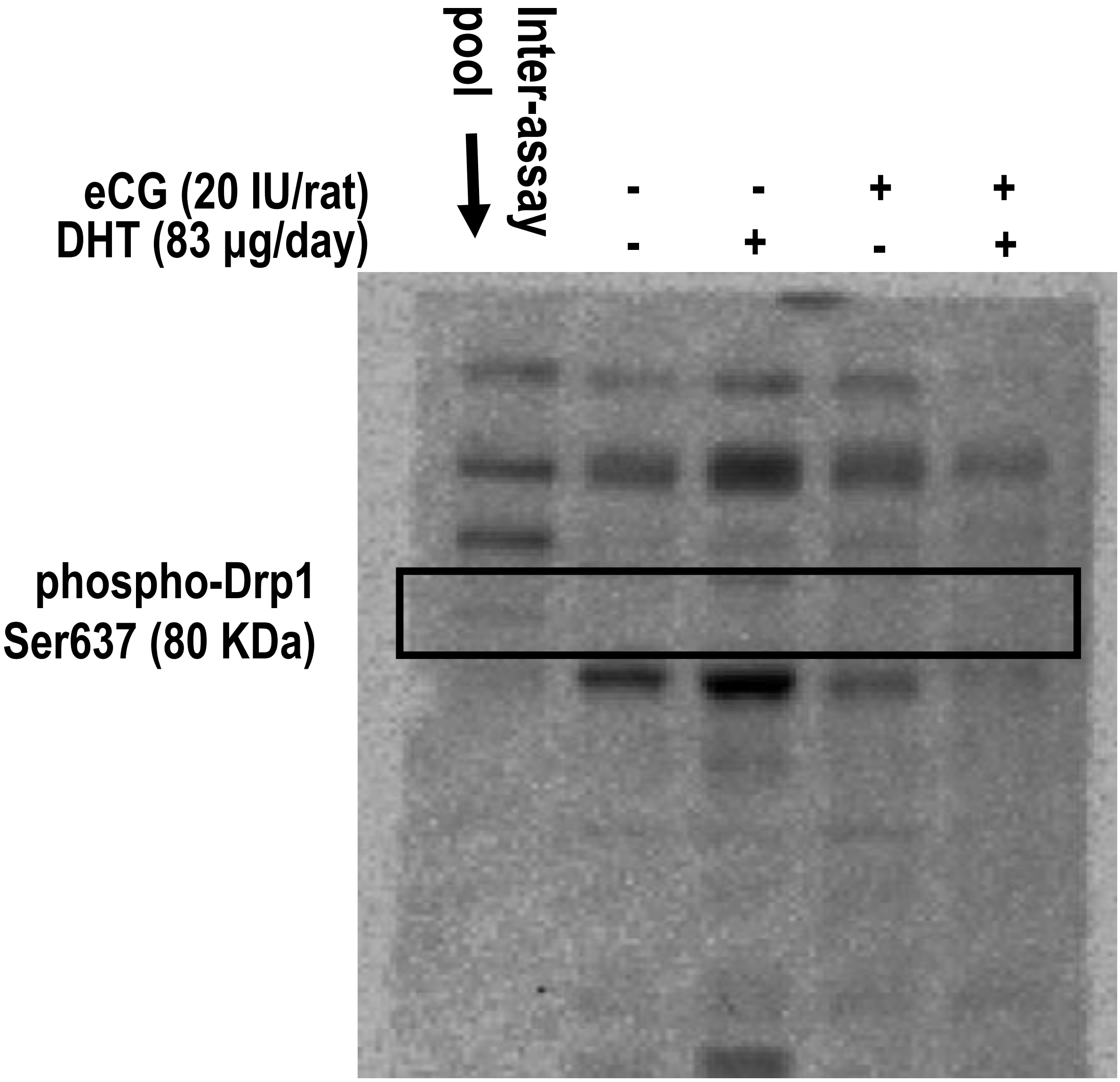
Inter-assay pool
eCG (20 IU/rat) - - + +
DHT (83 µg/day) - + - +
phospho-Drp1
Ser637 (80 KDa)

Fig. 7

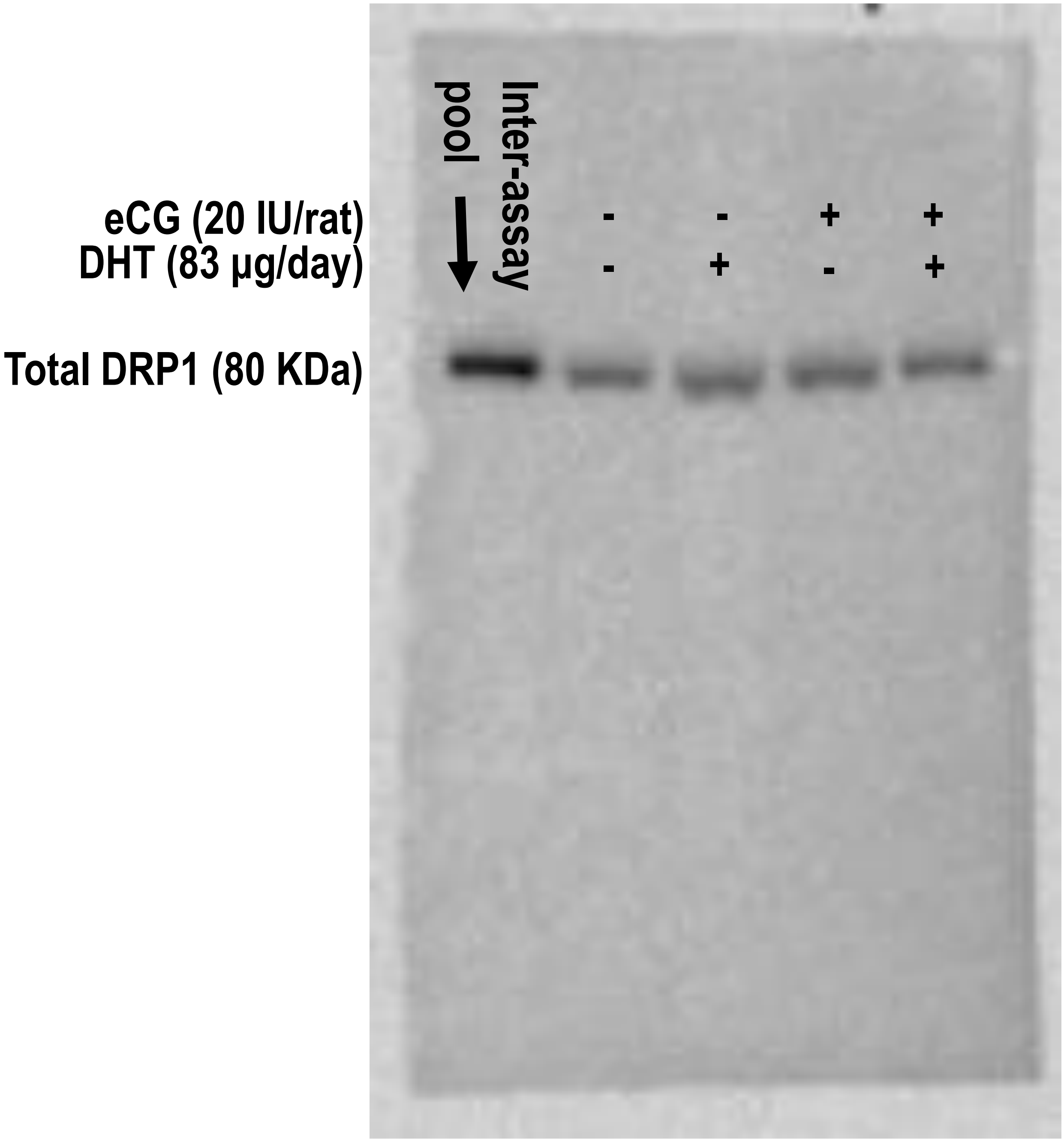

Fig. 7

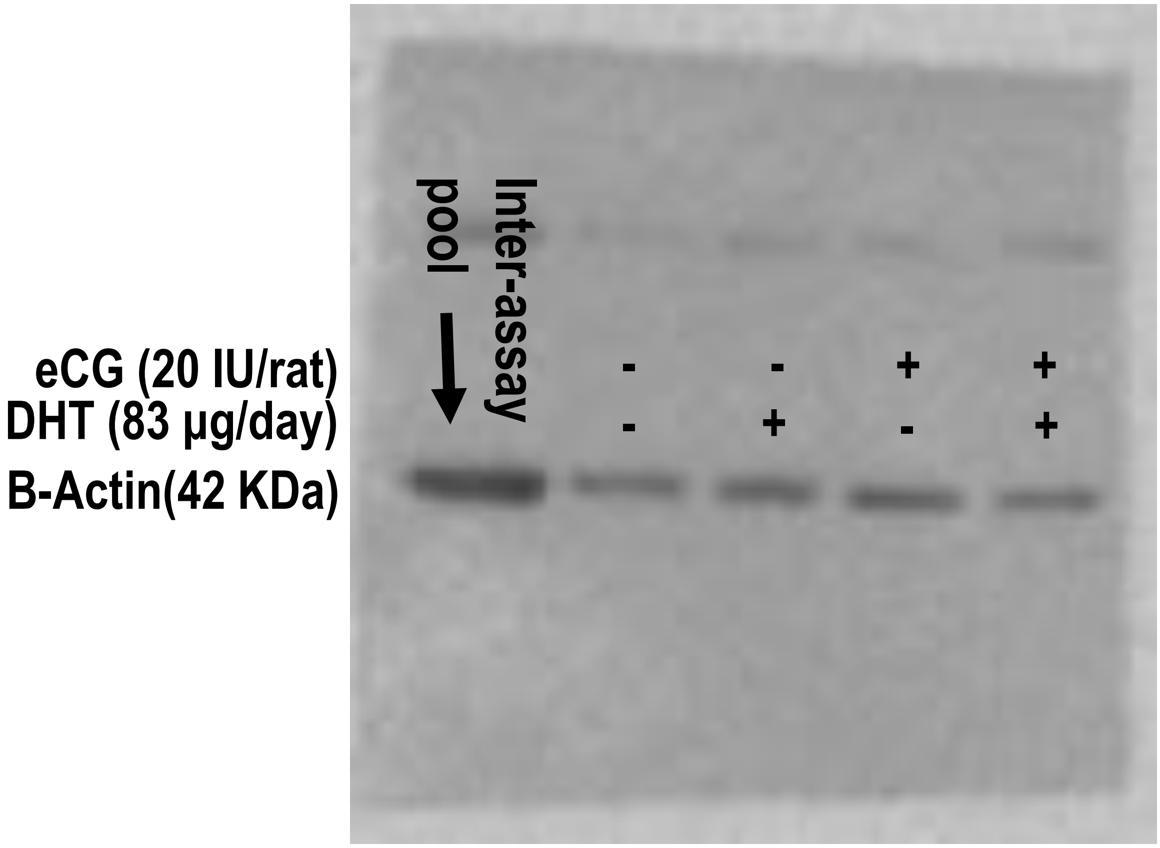

Supplement: Supplementary file 1 — Supplementary figures. [file 41598_2020_57672_MOESM1_ESM.pdf]
